# An Analysis of the Design and Kinematic Characteristics of an Octopedic Land–Air Bionic Robot

**DOI:** 10.3390/s25144502

**Published:** 2025-07-19

**Authors:** Jianwei Zhao, Jiaping Gao, Mingsong Bao, Hao Zhai, Xu Pei, Zheng Jiang

**Affiliations:** 1 Faculty of Mechanical and Electrical Engineering, China University of Mining & Technology-Beijing, Beijing 100083, China; 18015178726@163.com (H.Z.); a13765252515@163.com (Z.J.); 2 School of Mechanical Engineering and Automation, Beihang University, Beijing 100191, China; 15140831446@163.com (J.G.); peixu@buaa.edu.cn (X.P.); 3 Shandong Guoxing Intelligent Technology Co., Ltd., Yantai 264000, China; terrybao@suprobot.com

**Keywords:** wheel-legged robots, configuration design, wheel-foot composite, kinematic modeling, gait planning

## Abstract

The urgent need for complex terrain adaptability in industrial automation and disaster relief has highlighted the great potential of octopedal wheel-legged robots. However, their design complexity and motion control challenges must be addressed. In this study, an innovative design approach is employed to construct a highly adaptive robot architecture capable of intelligently adjusting the wheel-leg configuration to cope with changing environments. An advanced kinematic analysis and simulation techniques are combined with inverse kinematic algorithms and dynamic planning to achieve a typical ‘Step-Wise Octopedal Dynamic Coordination Gait’ and different gait planning and optimization. The effectiveness of the design and control strategy is verified through the construction of an experimental platform and field tests, significantly improving the robot’s adaptability and mobility in complex terrain. Additionally, an optional integrated quadrotor module with a compact folding mechanism is incorporated, enabling the robot to overcome otherwise impassable obstacles via short-distance flight when ground locomotion is impaired. This achievement not only enriches the theory and methodology of the multi-legged robot design but also establishes a solid foundation for its widespread application in disaster rescue, exploration, and industrial automation.

## 1. Introduction

Octoped wheeled-legged robots are increasingly attracting academic attention in the cutting-edge field of robotics due to their exceptional adaptability and flexibility in complex terrains. These robots represent an innovative combination of wheeled mobility and legged locomotion, offering novel solutions for task execution in complex environments. Liu, Xin, Xu, Kun et al. emphasize that in unpredictable disaster sites and extreme terrains, multi-legged, wheeled, and legged robots demonstrate superior mobility and stability compared to traditional wheeled or tracked mobility platforms [1,2,3,4]. Their outstanding obstacle-crossing capabilities and high adaptability distinguish them from other robotic systems.

As the demand for exploration in high-risk operational environments, such as nuclear power maintenance and disaster site search and rescue, continues to grow, the requirements for robot functionality are becoming increasingly stringent. However, traditional ground-based mobile robots (whether wheeled, tracked, or legged) often face fundamental limitations in mobility when encountering extremely complex, unstructured terrain (such as large-scale rubble, deep gullies, steep slopes, or water barriers). A single land-based mobility mode cannot meet the demands for a comprehensive coverage, rapid responses, and efficient operations. Therefore, an eight-legged wheeled robot must not only possess efficient ground mobility capabilities but also advanced capabilities in environmental perception, decision-making, and motion control.

**Figure 1:**
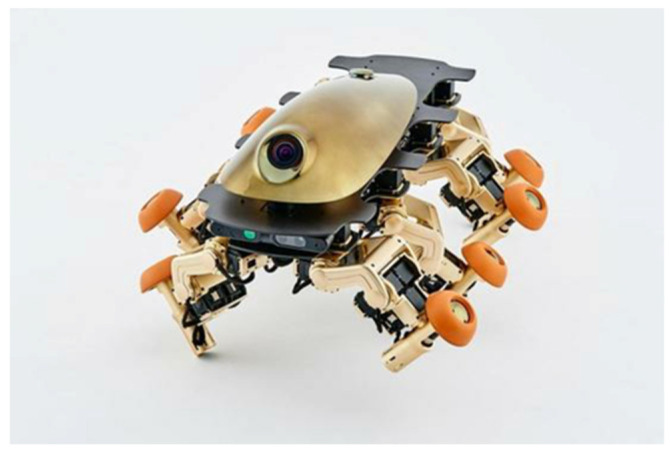
Halluc II robot.

Inspired by the unique ability of natural organisms to combine terrestrial walking and aerial flight, robotics research is actively exploring amphibious bio-inspired directions [5,6]. Amphibious robots can overcome the limitations of a single mobility mode by integrating flight capabilities, enabling them to switch to an aerial mode when encountering insurmountable ground obstacles or requiring rapid reconnaissance and the traversal of vast areas, thereby achieving a more comprehensive and flexible adaptation to complex operational conditions. For example, the M4 robot, jointly developed by the California Institute of Technology and Northeastern University, achieves multiple movement modes (including rolling, walking, and flying) by flexibly utilizing the same kinematic mechanism, significantly enhancing its mobility and environmental adaptability [7]. The Harbin Institute of Technology’s MTABot enhances the robot’s obstacle-crossing capability by optimizing the radius and sector angle of its deformable wheels [8]. Japan’s Halluc II (Figure 1) and its successor model Halluc IIx demonstrate a flexible switching between wheeled and octopod modes [9]. NASA’s ATHLETE [10] Osaka University’s ASTERISKH [11], and Beihang University’s E-beetle have all demonstrated their efficient performance in ground tasks. Tsinghua University’s novel wheel-legged mobile robot switches between locomotion modes via a clutch, demonstrating excellent adaptability and maneuverability [12]. These research advancements collectively point to a trend: integrating multiple locomotion modes, particularly land–air coordination, is an effective approach to enhancing adaptability in complex environments.

Based on the above research background, this paper focuses on the design of an octopedal robot and its kinematic analysis. Inspired by the land–air bionic concept—where natural creatures adapt to diverse environments through multi-modal locomotion—this robot integrates both high-performance ground mobility and an optional aerial capability, distinguishing it from existing systems like Halluc II.

Compared to Halluc II, the newly designed octopedal wheel-legged robot achieves a multi-height adaptive step climbing capability with its innovative Step-Wise Octopedal Dynamic Coordination Gait. Specifically, each leg is equipped with three active joints (one more than Halluc II’s two-joint structure), significantly expanding the motion range and enhancing the obstacle-crossing performance for terrains with 110–180 mm height variations.

Notably, an optional, integrated quadrotor module with a compact folding mechanism is introduced to enable land–air transitions. This module remains folded during ground locomotion to avoid interference, but deploys rapidly when ground movement is impaired, providing a short-distance flight capability. This dual-mode design—combining advanced wheel-leg locomotion and aerial mobility—addresses the limitations of single-terrain robots, offering a more comprehensive solution for complex scenarios such as disaster zones where both ground and air traversal may be required.

The effectiveness of the wheel-legged design and control strategy is verified through the construction of an experimental platform and field tests, significantly improving the robot’s adaptability and mobility in a complex terrain. This achievement not only enriches the theory and methodology of the multi-legged robot design but also establishes a solid foundation for its widespread application in disaster rescue, exploration, and industrial automation.

## 2. The Structural Design of an Octopedal Wheel-Legged Robot

### 2.1. Leg Mechanism Design Requirements

The design of the leg mechanism of an eight-legged wheeled legged robot is crucial, as its performance is directly related to the overall working efficiency of the robot [13,14,15]. In the design process, the following key requirements must be considered:

(a) Motion Requirements: The robot should be capable of performing forward and backward movements in a straight line, turning, ascending steps, adjusting leg spacing, raising and lowering the body, and switching between wheeled and footed modes while maintaining overall stability. The leg mechanism design must have a sufficient range of motion to meet the actual operational requirements.

(b) Weight Requirements: As the robot is driven by servos at the joints, a lightweight design of the leg structure is necessary to reduce the overall weight of the robot and ensure that the torque at the joints does not exceed the rated torque of the servos [16].

After actual measurements, the height of the steps in the teaching building and outdoor square was found to be between 110 mm and 180 mm. The preliminary robot functional parameters and dimensional parameters are shown in Table 1.

### 2.2. Leg Mechanism Design

**Figure 2:**
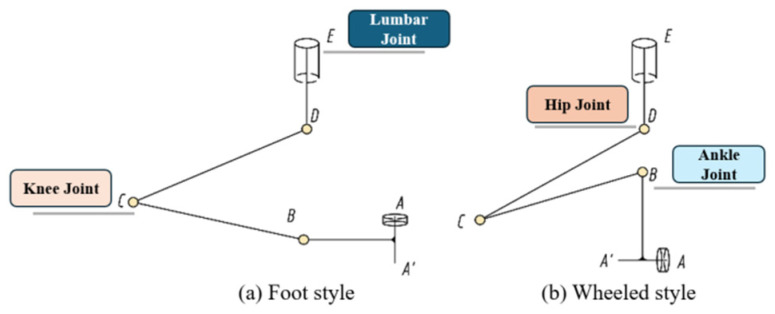
(**a**) Foot style; (**b**) Wheeled style. A sketch of a wheel-footed leg. E represents the hip joint axis connected to the body, while D, C, and B are the three active joints. A is the wheel of the leg, with its axis parallel to the ground when the robot is moving in the wheeled mode. When the robot is moving in the footed mode A′, the end of the foot touches the ground, enabling the robot to crawl on complex surfaces.

Each leg integrates three active joints—a critical advancement over Halluc II’s two-joint legs. This added degree of freedom expands the kinematic workspace (Section 3.1) and enables dynamic gait coordination for higher obstacles. The sketch of the leg structure for both wheeled and footed walking modes is shown in Figure 2:

### 2.3. Overall 3D Modeling of the Robot

**Figure 3:**
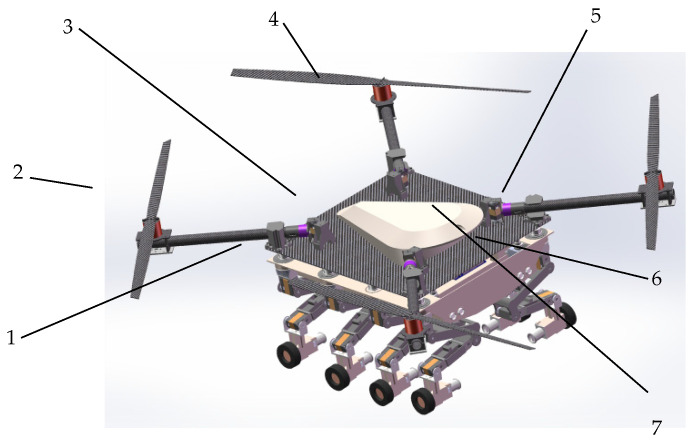
The model diagram of the whole octopedal wheel-legged robot. 1. Carbon Board. 2. Paddle. 3. Upper Shell. 4. Brushless Motor. 5. Carbon Tube. 6. Fuselage. 7. Folding Servo.

The designs of the leg mechanism, layout, and dimensional parameters are the main issues in the design of a wheeled-legged robot. These aspects need to be comprehensively considered in terms of the robot’s stability, energy efficiency, and economy [17,18]. In this paper, the leg mechanism of the wheel-legged robot is designed according to the application and motion needs of the robot. The robot’s overall structural model, as shown in Figure 3, mainly consists of eight leg mechanisms of the same structure, the body 6, the carbon plate 1, and the upper shell 3. Four rotors are installed on the carbon plate to provide the robot with the ability to fly. The folding servo 7 drives the rotors to unfold when in flight mode and retracts them when moving on the ground, making the robot compact. This design has a significant advantage, especially for tasks that require traversing narrow spaces or avoiding obstacles. It also reduces energy consumption and improves safety, helping to minimize potential injuries to people or objects during the robot’s operation.

**Figure 4:**
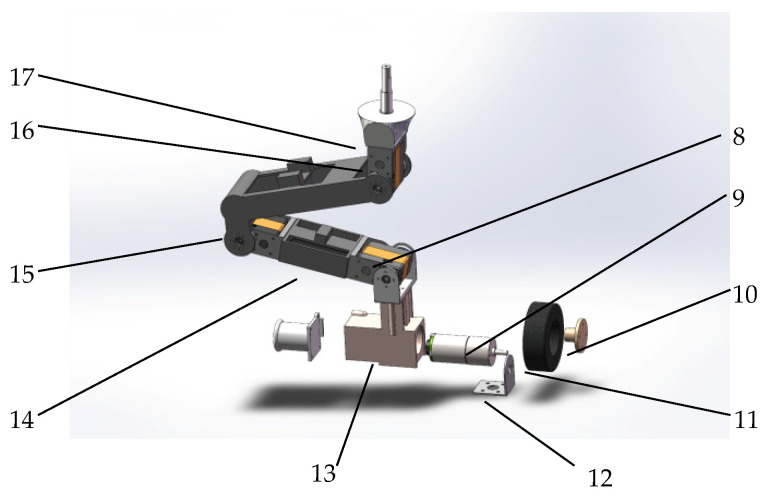
A modelled view of the leg structure. 8. articulating servo. 9. DC motor. 10. Coupling. 11. Wheel. 12. Motor mount. 13. Foot end. 14. Calf. 15. Thigh 16. U-bracket. 17. Hip joint.

The eight leg mechanisms of the robot have the same structure, and the leg mechanism configuration is designed as shown in Figure 4. The main structure consists of joint servos 8, DC motors 9, U-shape brackets 16, thighs 15, calves 14, foot ends 13, wheels 12, and other components. The three servos on each leg drive the joints to rotate, changing the robot’s attitude, while the DC motors on the foot end drive the robot’s movement.

**Figure 5:**
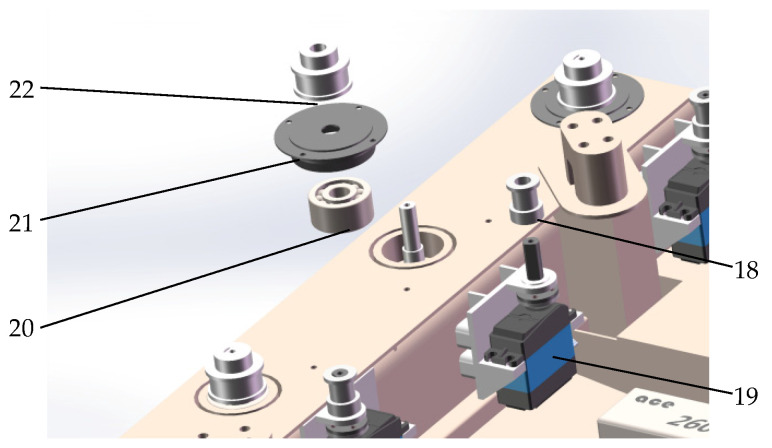
Steering mechanism diagram. 18. Small Synchronous Belt. 19. Steering Servo. 20. Bearing. 21. End Cap. 22. Large Synchronous Wheel.

The body is also equipped with eight steering servos, which drive the rotation of each leg through synchronous belt wheels, as shown in Figure 5. Since the robot is driven by eight steering servos, twenty-four joint servos, and eight DC motors, it can achieve a variety of complex actions, greatly improving its ability to cross obstacles and maintain stability.

## 3. The Leg Mechanism of an Octopedal Wheeled-Legged Robot: A Core Research Analysis of Motion and Statics

### 3.1. The Positional Analysis of the Leg Mechanism

**Figure 6:**
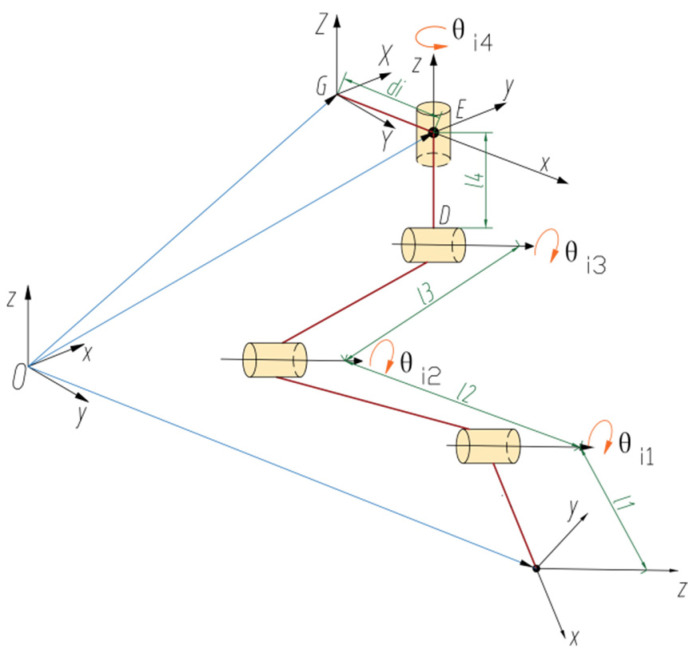
A sketch of the leg lifting mechanism.

The walking control of the octopedal wheel-legged robot is primarily dependent on its kinematic planning, with an initial analysis of the forward motion characteristics of the lifting leg. As illustrated in Figure 6, the diagrammatic representation of the mechanism depicts the configuration of the lifting leg. To solve the forward kinematics of the lifting leg, four coordinate systems are initially established: the global coordinate system $\left\{O\right\}$, the fuselage coordinate system $\left\{G\right\}$, the hip coordinate system $\left\{E_{i}\right\}$, and the foot coordinate system $\left\{A_{i}\right\}$. The global coordinate system $\left\{O\right\}$ is based on the robot’s environment; the robot itself has a fuselage system $\left\{G\right\}$, whose origin coincides with the geometric center of the robot. The z-axis of the hip coordinate system $\left\{E_{i}\right\}$ coincides with the axis of the rotation of the hip joint, and the origin is fixed to the body. The origin of the foot coordinate system $\left\{A_{i}\right\}$ is coincident with the foot, and the x-axis is along the direction of the lower leg, setting the layout, where the robot leg reaches the fully extended state as the starting configuration. The coordinate system parameters are shown in Figure 6. Since both the coordinate system and the waist joint coordinate system are cemented to the robot body, it is possible to move from the coordinate system $\left\{G\right\}$ to the coordinate system $\left\{E_{i}\right\}$. The transformation matrix is also fixed and can be described as

(1) gGEi=REiGPEiG01

Equation: REiG represents the 3 × 3 rotation matrix, given by

(2) REiG=cosθi5−sinθi50sinθi5cosθi50001

$\theta_{i5}$ represents the angle of the rotation of the lumbar joint relative to the fuselage about the z-axis.

PEiG represents the 3×1 the position vector of

(3) PEiG=dxdy0

where $d_{x}$ and $d_{y}$ represent the distances from the center of the fuselage to the hip joint in the x-direction and y-direction, respectively.

In the starting configuration, the transformation matrix from the coordinate system $\left\{E_{i}\right\}$ to the coordinate system $\left\{A_{i}\right\}$ is

(4) $$g_{E_{i}A_{i}}\left(0\right)=\left[\begin{array}{cc} I_{3\times 3} & \begin{array}{c} 1\\ 1\\ l_{1}+l_{2}+l_{3}+l_{4} \end{array}\\ 0 & 1 \end{array}\right]$$

The length of each leg segment is defined as follows: foot end length $l_{1}$, calf length $l_{2}$, thigh length $l_{3}$, and crotch length $l_{4}$. Kinematic rotation $\xi_{ij}$ describes the length of the joint of the leg, and the rotational motion of each rotational joint can be expressed as j. The rotational motion of each rotational joint can be expressed as

(5) $$\xi_{ij}=\left[\begin{array}{c} \omega_{ij}\\ r_{ij}\times \omega_{ij} \end{array}\right]$$

The direction vector of the rotation axis is defined as ωij=(ωijxωijyωijz)T, where each component represents the projection of the rotation axis in the X, Y, and Z directions, respectively. The vector of any point on the rotation axis is denoted as rij=(rijxrijyrijz)T, where the vector points from the origin to the point on the axis.

Thus, the positive kinematics of lifting the leg can be expressed in terms of the exponential product equation as follows:

(6) $$g_{{OA}_{i}}\left(\theta \right)=g_{OG}g_{{GE}_{i}}e^{{\theta_{i1}}_{\xi_{i1}}}e^{{\theta_{i2}}_{\xi_{i2}}}e^{{\theta_{i3}}_{\xi_{i3}}}e^{{\theta_{i4}}_{\xi_{i4}}}g_{E_{i}A_{i}}\left(0\right)$$

where $\theta_{i4}$ represents the lumbar joint variable; $\theta_{i3}$ represents the hip joint variable; $\theta_{i2}$ represents the knee joint variable; $\theta_{i1}$ represents the foot joint variable; and $g_{OG}$ represents the transformation matrix from the global coordinate system $\left\{O\right\}$ to the fuselage coordinate system $\left\{G\right\}$.

(7) gOG=RGOPGO01

where RG O represents a 3 × 3 rotation matrix representing the pose of the robot body in the global coordinate system, and PG O represents a 3 × 1 position vector representing the position of the robot body in the global coordinate system. The foot coordinate system $\left\{A_{i}\right\}$ in the global coordinate system $\left\{O\right\}$ is Equation (8).

(8) $$\left[\begin{array}{cc} \begin{array}{cc} c\theta_{i1}\theta_{i2}\theta_{i3}c\theta_{i4}\theta_{i5} & -c\theta_{i4}\theta_{i5}s\theta_{i1}\theta_{i2}\theta_{i3}\\ c\theta_{i1}\theta_{i2}\theta_{i3}s\theta_{i4}\theta_{i5} & -s\theta_{i4}\theta_{i5}s\theta_{i1}\theta_{i2}\theta_{i3} \end{array} & \begin{array}{cc} s\theta_{i4}\theta_{i5} & c\theta_{i4}\theta_{i5}\left(l_{i1}c\theta_{i1}+l_{i2}c\theta_{i2}+l_{i3}c\theta_{i3}\right)+d_{xi}\\ -c\theta_{i4}\theta_{i5} & s\theta_{i4}\theta_{i5}\left(l_{i1}c\theta_{i1}+l_{i2}c\theta_{i2}+l_{i3}c\theta_{i3}\right)+d_{yi} \end{array}\\ \begin{array}{cc} s\theta_{i1}\theta_{i2}\theta_{i3} & c\theta_{i1}\theta_{i2}\theta_{i3}\\ 0 & 0 \end{array} & \begin{array}{cc} 0 & l_{i1}s\theta_{i1}+l_{i2}s\theta_{i2}+l_{i3}s\theta_{i3}+l_{i4}\\ 0 & 1 \end{array} \end{array}\right]$$

The transformation matrix in the dimensions of the robot and the joint rotation angle limits are shown in Table 2.

**Figure 7:**
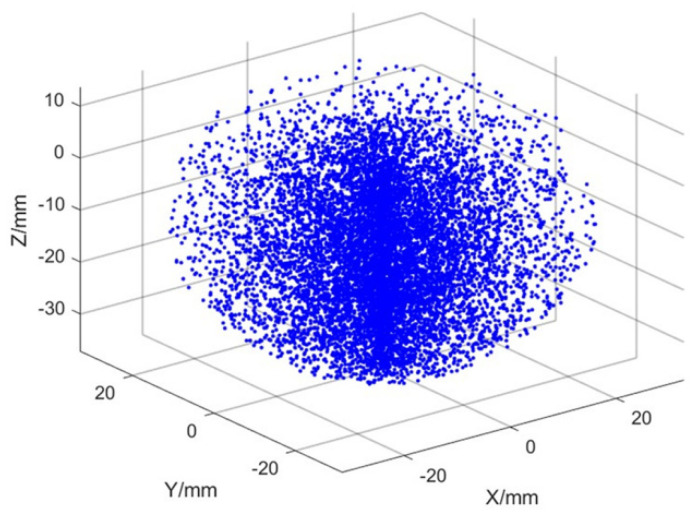
One-legged reachable movement space.

Based on the positive kinematic model of the robot’s single leg and its main parameters, the reachable workspace of the robot’s single leg can be obtained by a simulation, as shown in Figure 7.

**Figure 8:**
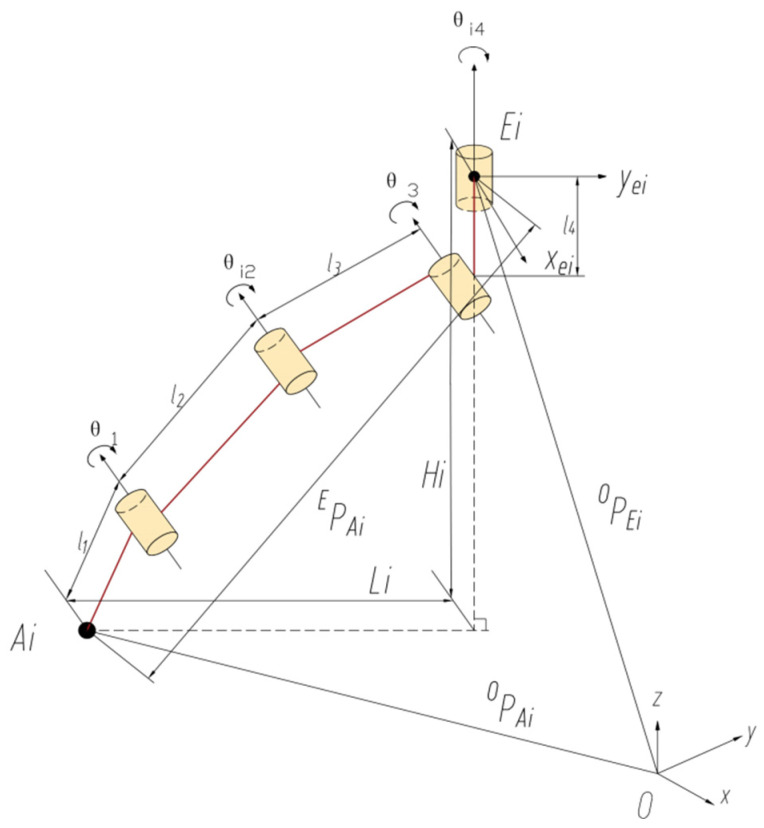
A sketch of the i-th leg space mechanism.

The ground motion attitude of the robot is achieved by controlling the drive joints of all legs, so it is necessary to solve the angles of the drive joints of all legs through the inverse kinematics of the robot. A sketch of the spatial mechanism of the ith leg is shown in Figure 8. In the figure the PEio, REio, and PAio are given, while the drive joint variables $\theta_{i1}$,${\;\theta }_{i2}$, $\theta_{i3}$, and $\theta_{i4}$ are unknown.

The position vector of the reference point A at the foot end is set as ${\left(x,y,\;z,\alpha ,\beta ,\gamma \right)}^{T}$, where ${\left(x,y,z\right)}^{T}$ denotes the position coordinates of the foot end reference point in the fixed coordinate system E−xyz, and ${\left(\alpha ,\beta ,\gamma \right)}^{T}$ denotes the attitude coordinate of the foot end reference point relative to the fixed coordinate system E−xyz. The α in the position vector represents the rotation vector of the moving coordinate system around the x-axis of the fixed coordinate system, which is the amplitude of the leg swing; that is, the angle of rotation of the stride; β denotes the rotation vector of the moving coordinate system around the y-axis of the fixed coordinate system; and γ denotes the rotation vector of the moving coordinate system around the z-axis of the fixed coordinate system. From the leg mechanism, it is known that $\beta =0,\gamma =\theta_{4}$_._ The coordinate mapping can be used to describe the position of the foot end movement in the fixed coordinate system. Based on the structural characteristics of the leg mechanism, it is known that three joint rotation angles $\theta_{1\;,\;}\theta_{2},\;\theta_{3},$ and posture α are interrelated, and their geometric relationships are as follows:

(9) $$\alpha =\theta_{1}+\theta_{2}{+\theta }_{3}$$

According to the transformation relationship of vectors between coordinate systems, the following equation is obtained:

(10) PAio=PEio+REioPAiE

This gives us

(11) PAiE=ROEiPAio−PEio=R−1EioPAio−PEio

In the plane where the axes of the calf, thigh, and lumbar joints lie, and in the plane of the body perpendicular to the axis of the lumbar joints, the geometrical relationship shown in Equation (12) exists.

(12) $$\left\{\begin{array}{c} L_{i}=L_{3}\sin\theta_{3}+L_{2}\sin{(\theta_{2}+\theta }_{3})\\ +L_{1}\sin\left(\theta_{1}+\theta_{2}+\theta_{3}\right)\\ H_{i}=L_{4}+L_{3}\cos\theta_{3}+L_{2}\cos{(\theta_{2}+\theta }_{3})\\ +L_{1}\cos\left(\theta_{1}+\theta_{2}+\theta_{3}\right) \end{array}\right.$$

Based on the geometric relationship shown in Figure 8, the following equations can also be obtained:

(13) Li=xAi2E+yAi2EHi=−zAiEtanθ4=xAiEyAiE       yAiE≥0

(14) Li=−xAi2E+yAi2EHi=−zAiEtanθ4=xAiEyAiE       yAiE<0

where (xAiEyAiE zAiE) represents the foot endpoint $A_{i}$ in the coordinate system ${\left\{E\right\}}_{i}$. If the foot endpoint $A_{i}$ is known in the coordinate system {O} and the body’s position in the coordinate system {O} is given, the joint angle variables $\theta_{1\;},\theta_{2},\theta_{3},and\;\theta_{4}$ can be derived according to Equations (5)–(9).

### 3.2. Solving the Velocity Relation Matrix

The displacement of the reference point at the end of the leg mechanism is realized by three drives. The symbolic expression between the input and output obtained by the position inverse solution above is derived here to obtain the velocity transfer relation of the leg mechanism, where the moving platform is the reference point at the end of the foot. The velocity mapping relationship between the output and input of the mechanism is given by

(15) v=J·q

where v represents the output velocity of the reference point at the foot end in a fixed coordinate system.

$$v={\left[\dot{x_{A}}\dot{{,y}_{A}}\dot{{,z}_{A},}\dot{\alpha }\right]}^{T}$$

J denotes the matrix of the output and input velocity transfer relationship of the leg mechanism, q signifies the output speed of the leg servo driver, and the $q={\left[\dot{\theta_{i1}},\dot{\theta_{i2}},\dot{\theta_{i3}},\dot{\theta_{i4}}\right]}^{T}$.

From the positional inverse solution of the leg mechanism above, it follows that by replacing Equation (9) with Equations (11)–(14), an expression for the angle of the drive joint in the fixed coordinate system with respect to the reference point at the foot end is obtained:

(16) $$\theta_{j}=f\left(x_{A},y_{A},z_{A},\alpha \right)$$

Both sides can simultaneously take the derivative of time t to obtain

(17) $$\dot{\theta_{j}}=\left[\frac{\partial f_{j}}{\partial x_{A}}\frac{\partial f_{j}}{\partial y_{A}}\frac{\partial f_{j}}{\partial z_{A}}\frac{\partial f_{j}}{\partial \alpha }\frac{\partial f_{j}}{\partial \gamma }\right]\left[\begin{array}{c} \begin{array}{c} \dot{x_{A}}\\ \dot{y_{A}}\\ \dot{z_{A}} \end{array}\\ \begin{array}{c} \dot{\alpha }\\ \dot{\gamma } \end{array} \end{array}\right]$$

(18) q=G·v

of which $G=\left[\frac{\partial f_{j}}{\partial x_{A}}\;\frac{\partial f_{j}}{\partial y_{A}}\;\frac{\partial f_{j}}{\partial z_{A}}\;\frac{\partial f_{j}}{\partial \alpha }\;\frac{\partial f_{j}}{\partial \gamma }\right]$ and j=1, 2, 3, 4. Thus, G is the velocity mapping relationship between the input and output, and the deformation of Equation (19) leads to

(19) $$v=G^{-1}\cdot q$$

So far, the velocity mapping relation of the robot leg mechanism is obtained, and $G^{-1}=J$, which is the velocity Jacobi matrix of the leg mechanism designed in this paper.

## 4. Gait Strategies for Octopedal Wheel-Legged Robots

The gait design of an eight-legged robot directly affects the smoothness of the whole machine, the traveling speed, and the amount of driving torque required by the joints during the robot’s walking process [19,20,21,22,23]. The robot gait is divided into two main parts: the wheeled motion with wheels contacting the ground and the legged locomotion with the end of the foot contacting the ground.

### 4.1. Wheeled Motion Mode

**Figure 9:**
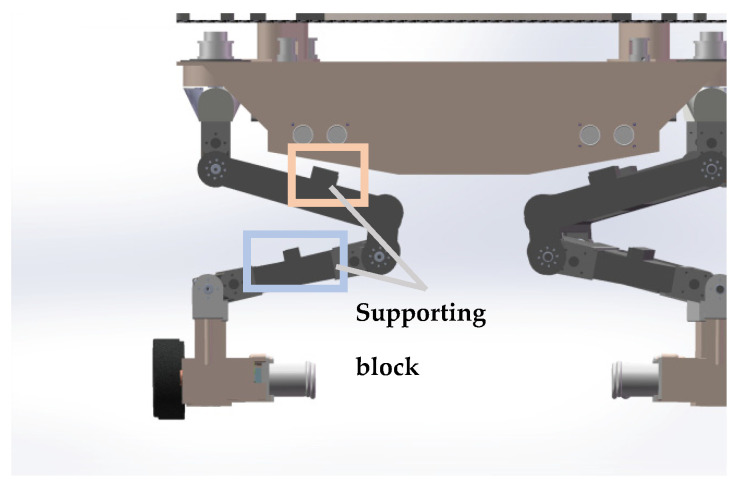
A demonstration of the attitude of a flat-floor walking robot.

When the robot is wheeling on a flat, unobstructed surface, all eight wheels are in contact with the ground simultaneously, and the robot’s legs remain folded, as shown in Figure 9. The thighs and calves are kept parallel to the side of the fuselage, and the bottom of the fuselage is closely attached to the bottom in sequence. The ends of the foot are kept upright, which greatly reduces the load on servos 2 and 3 and reduces energy consumption. At the same time, the center of gravity of the body is lowered to improve the stability of the robot.

**Figure 10:**
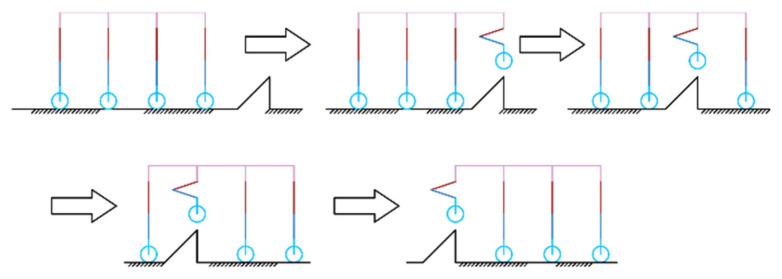
Small obstacle-crossing gait.

When encountering potholes or obstacles, the robot uses infrared scanning to determine whether it can cross the obstacles and calculate the height to which the foot end needs to be lifted. Take a small protruding obstacle (width less than the leg spacing, height lower than the maximum height of the obstacle) as an example, and design the obstacle-crossing gait, as shown in Figure 10. The obstacle-crossing gait consists of the following steps: (1) Before crossing the obstacle, the robot adjusts its attitude, and the eight legs are distributed symmetrically in a straight-line form on both sides of the machine body. (2) Upon arriving at the front of the obstacle, L1 and R1 are lifted, while the remaining six legs serve as support items and drive the wheels forward. (3) After L1 and R1 cross the obstacle, they return to the straight-line form. L2 and R2 are then lifted, with the remaining six legs serving as support items and driving the wheels forward. In this manner, the legs are made to cross the obstacles in turn.

**Figure 11:**
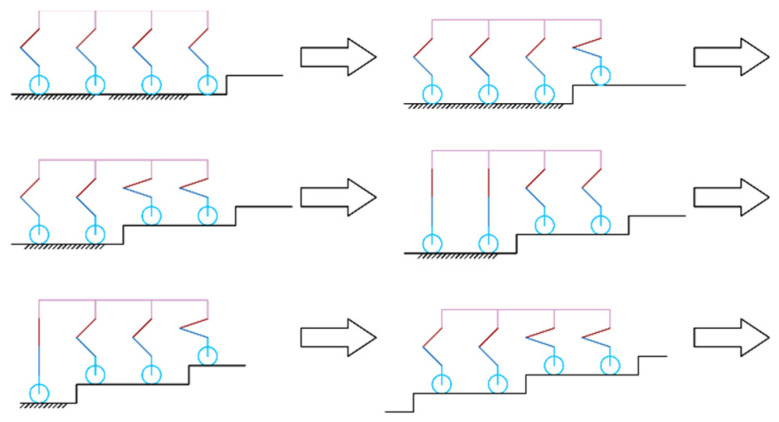
Step-up gait.

The robot’s ‘Step-Wise Octopedal Dynamic Coordination Gait’ is accomplished in six main steps, as shown in Figure 11:

(a) Before going up the steps, the robot adjusts its attitude and lifts the fuselage by a height of h+δ from the contracted state, where is the height of one step, and δ is the height allowance of the foot end above the step surface when the foot end is raised to its highest point.

(b) L1 and R1 are lifted to a distance of δ above the first step, while the remaining six legs serve as support items and drive the wheels forward. When L1 and R1 reach above the step surface, the legs are extended so that the foot ends are in contact with the step surface.

(c) L2 and R2 are lifted to a distance of δ above the first step, while the remaining six legs serve as support items and drive the wheels forward. When L2 and R2 reach above the step surface, the legs are extended so that the foot ends are in contact with the step surface.

(d)–(e) Since the first and second rows of legs are in a contracted state, they cannot continue to lift the next step. Therefore, all eight legs are lifted simultaneously by a height of h, and L1, R1, L3, and R3 continue to be lifted, with the remaining four legs serving as support items and driving the wheels forward. When L1, R1, L3, and R3 reach the top of the step surface, the legs are stretched out so that the ends of the feet are in contact with the step surface.

(f) L2, R2, L4, and R4 are lifted to the next step. At this time, the gait in (c) and (f) is the same, so by reciprocating (c)–(f), multi-step climbing can be achieved.

**Figure 12:**
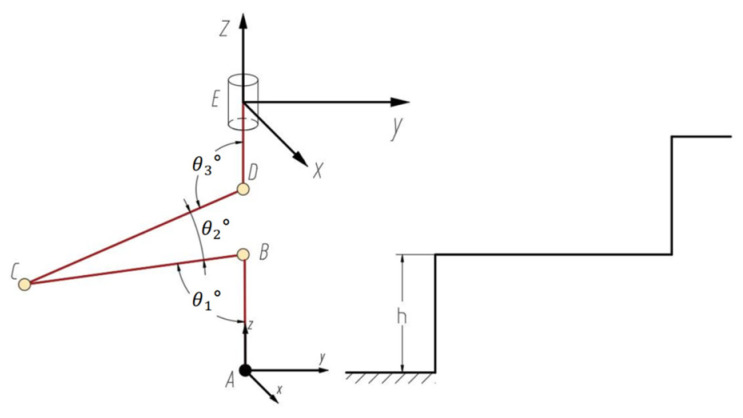
A sketch of the step-traveling leg mechanism.

Since going up the steps primarily involves changing $z_{A}$, to simplify the gait, make $x_{E}=x_{A}\;and{\;y}_{E}=y_{A}$. As shown in Figure 12, at this point, the three joints satisfy the following relationship:

(20) $$\frac{l_{CD}}{sin\theta_{1}}=\frac{l_{DB}}{sin\theta_{2}}=\frac{l_{BC}}{sin\theta_{3}}$$

Derive $\theta_{1}$ and the linear relationship between $\theta_{2}\;and\;\theta_{3}$.

When the eight-legged robot steers, the robot rotates around a point to achieve steering. The orientation controller is designed based on the deviation between the current position of the robot and the target position. The deviation and the rate of change in the deviation are used as inputs to the fuzzy controller, and the control quantity is the rotation angle of the wheel-legs. The rotation angle of each wheel-leg is obtained through kinematic modeling to achieve orientation control.

As for the direction control of this robot, firstly, when the eight-legged robot is steering, based on the structural advantages of the independent drive and the independent steering of the eight wheels, the robot can control the front and rear wheels to rotate by the corresponding angle simultaneously during the steering process of the wheel motion, so as to obtain a larger steering angle with a smaller steering radius. The steering angle of the wheels follows the Ackermann steering principle.

**Figure 13:**
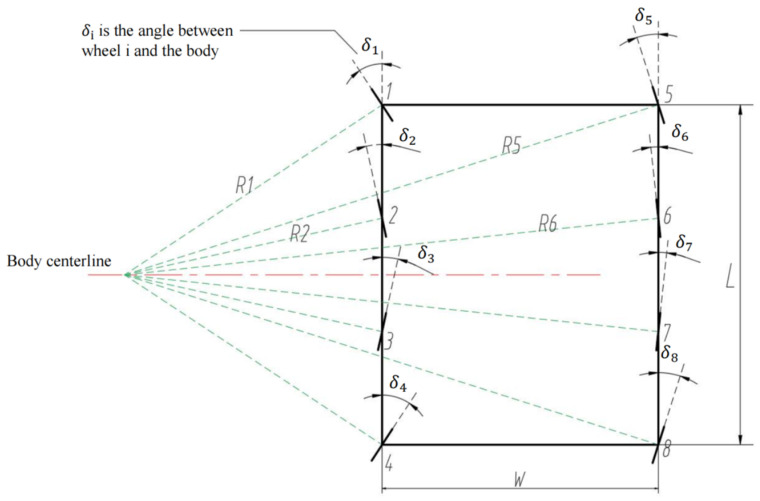
Robot 8-wheel steering mechanism sketch.

A robot steering kinematics model is established in the following, as shown in Figure 13. Eight legs are laid out equidistantly, with four on each side of the left and right sides, which are labeled as 1, 2, 7, and 8. The distance between the front and rear wheel axes is L, and the distance between the left and right wheel axes is W. During the steering process, the eight wheels are controlled independently, and at a certain moment, all the wheels are in a circular motion around the same instantaneous center. The instantaneous angle of the rotation of each wheel is recorded as $\delta_{1},\delta_{1},\ldots ,\delta_{7},\delta_{8}$, and the steering radius of each wheel relative to the instantaneous center is $R_{1},R_{2},\ldots ,R_{7},R_{8}$.

Based on the angular geometry of the individual wheel steering, the following equations can be obtained:

(21) $$\sin\delta_{1}=\frac{L}{2R_{1}}$$

(22) $$\sin\delta_{2}=\frac{L}{6R_{2}}$$

(23) $$\sin\delta_{6}=\frac{L}{6R_{6}}$$

(24) $$\sin\delta_{5}=\frac{L}{2R_{5}}$$

Then, based on the instantaneous steering radius relationship of each wheel, the following equations can be obtained:

(25) $$R_{2}cos\delta_{2}-R_{1}cos\delta_{1}=0$$

(26) $$R_{6}cos\delta_{6}-R_{1}cos\delta_{1}=W$$

(27) $$R_{5}cos\delta_{5}-R_{1}cos\delta_{1}=W$$

From Equation (21) we have $R_{1}=\frac{L}{2sin\delta_{1}}$, and from Equation (22) can be obtained by $R_{2}=\frac{L}{6sin\delta_{2}}$ and substituting this into Equation (25). The joint solution can be obtained as $tan\delta_{1}=3tan\delta_{2}.$ This gives

(28) $$\delta_{2}=\frac{1}{3}arctan\delta_{1}$$

Similarly, by Equations (21), (23) and (26), the joint solution can be obtained as

(29) $$\delta_{6}=arctan\frac{Ltan\delta_{1}}{3L+6Wtan\delta_{1}}$$

Similarly, by Equations (21), (24) and (27), the joint solution can be obtained as

(30) $$\delta_{5}=arctan\frac{Ltan\delta_{1}}{L+2Wtan\delta_{1}}$$

Then, based on symmetry, the angles of the other four wheels can be obtained as

(31) $$\delta_{3}{=-\delta }_{2}$$

(32) $$\delta_{4}{=-\delta }_{1}$$

(33) $$\delta_{8}{=-\delta }_{5}$$

(34) $$\delta_{7}{=-\delta }_{6}$$

In this way, taking the turning angle $\delta_{1}$ of wheel No. 1 as a reference, it is possible to calculate the steering angle of each of the remaining wheels in the instantaneous angle of the turn during the process.

During robot steering, the instantaneous angular velocity of the rotation of each wheel around the steering center is the same, which can be obtained as

(35) $$\frac{v_{1}}{R_{1}}=\frac{v_{2}}{R_{2}}=\frac{v_{3}}{R_{3}}=\frac{v_{4}}{R_{4}}=\frac{v_{5}}{R_{5}}=\frac{v_{6}}{R_{6}}=\frac{v_{7}}{R_{7}}=\frac{v_{8}}{R_{8}}$$

Therefore, using the rotational speed of wheel No. 1$v_{1}$ as a reference, the rotational speed of each wheel during the robot’s steering process can be calculated, enabling the steering control of the robot.

The objective of velocity control is to maintain a stable velocity for the robot during steering. The velocity controller is designed based on the deviation between the robot’s current velocity and the target velocity. The deviation and its rate of change are utilized as inputs to the controller, with the control quantity being the rotational speed of the wheel-legs. By employing the aforementioned calculation, the rotational speed of each wheel-leg is obtained to achieve velocity control.

### 4.2. Wheel-Leg Motion Mode Switching Planning

The core design principle for the wheel-leg transition is to perform mode switching on a relatively flat terrain. This strategy is driven by

(1) Stability Assurance**: Flat surfaces provide stable support for the posture adjustment (e.g., lifting legs for wheel/foot-end switching), mitigating overturning risks on rough terrain;
(2) Actuator Protection**: Joint movements during transitions experience more predictable loads on even ground, reducing the impact damage;
(3) Energy Efficiency**: Eliminating the compensation for terrain irregularities simplifies the control and lowers the energy consumption;
(4) Transition Buffer**: Switching to the walking mode on flat areas before entering rough terrain, or reverting to the wheeling mode after exiting, ensures an optimal configuration for environmental adaptation.

**Figure 14:**
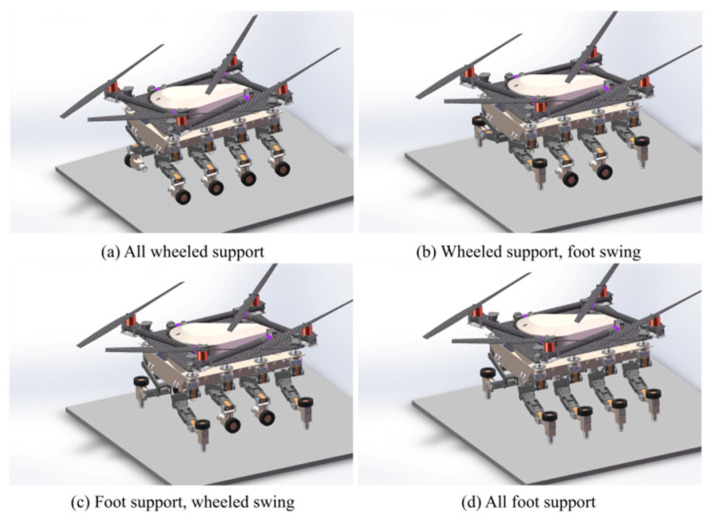
(**a**) All wheeled support; (**b**) wheeled support, foot swing; (**c**) Foot support, wheeled swing; (**d**) All foot support. Wheeling to walking motion mode switching planning.

The wheel-leg motion mode switching planning encompasses two types: transitioning from walking to wheeling and transitioning from wheeling to walking. These two types are inverse processes of each other. The process of switching from walking to wheeling is illustrated in Figure 14. In Figure 14a, all eight wheels of the robot simultaneously make contact with the ground in a supported state. Figure 14b depicts the robot entering the switching process, where four wheels (L2, R2, L3, and R3) support the body, while the remaining four wheeled legs lift and switch their foot ends for contact. In Figure 14c, L1, R1, L4, and R4 serve as support items, and the four legs L2, R2, L3, and R3 lift and switch their foot ends for contact. Finally, Figure 14d shows the robot completing the transition from walking mode to wheeling mode, with all eight leg foot ends touching the ground.

The inverse process of the wheeling-to-walking motion mode switching planning is depicted in Figure 14, from (d) to (a), corresponding to the walking-to-wheeling motion mode switching planning.

### 4.3. Foot Movement Patterns

**Figure 15:**
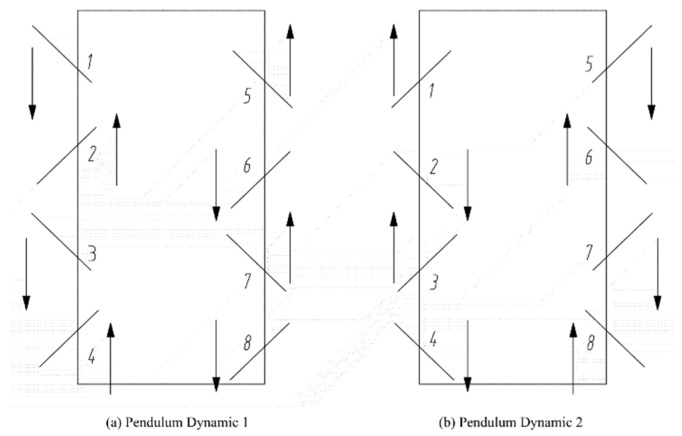
(**a**) Pendulum Dynamic 1; (**b**) Pendulum Dynamic 2. Foot gait planning.

The gait motion of the octopedal robot is illustrated in Figure 15. Legs 1, 3, 6, and 8 and legs 2, 4, 5, and 7 are selected as two sets of gaits to form the quadrilateral gait of the body.

Among the various gaits, the waveform gait is considered the most efficient and stable [24]. In the waveform gait, different strides are presented according to the load factor, which refers to the ratio of the support time of each walking leg in relation to the entire walking cycle, as shown in Equation (36).

(36) β=t/T

**Figure 16:**
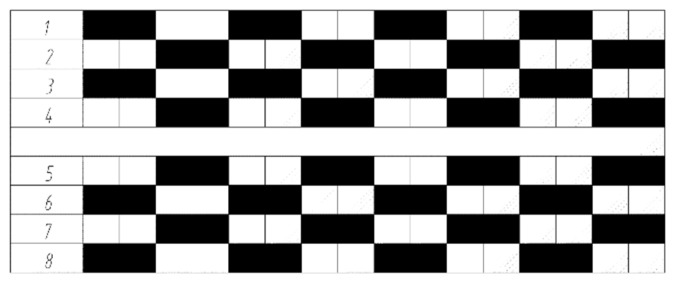
The timing diagram of the footed gait of an octopedal robot.

When the load factor is β=0.5, it represents the fastest statically stable gait that can be adopted by an eight-legged walking robot, known as the bi-quadruped gait [25]. In any walking cycle, two walking feet on both sides of the robot are in a supported state, allowing the walking robot to maintain good stability. The waveform timing diagram of the gait (the shaded part represents the suspended phase of the walking foot, and the blank part represents the supported phase of the walking foot) is shown in Figure 16.

**Figure 17:**
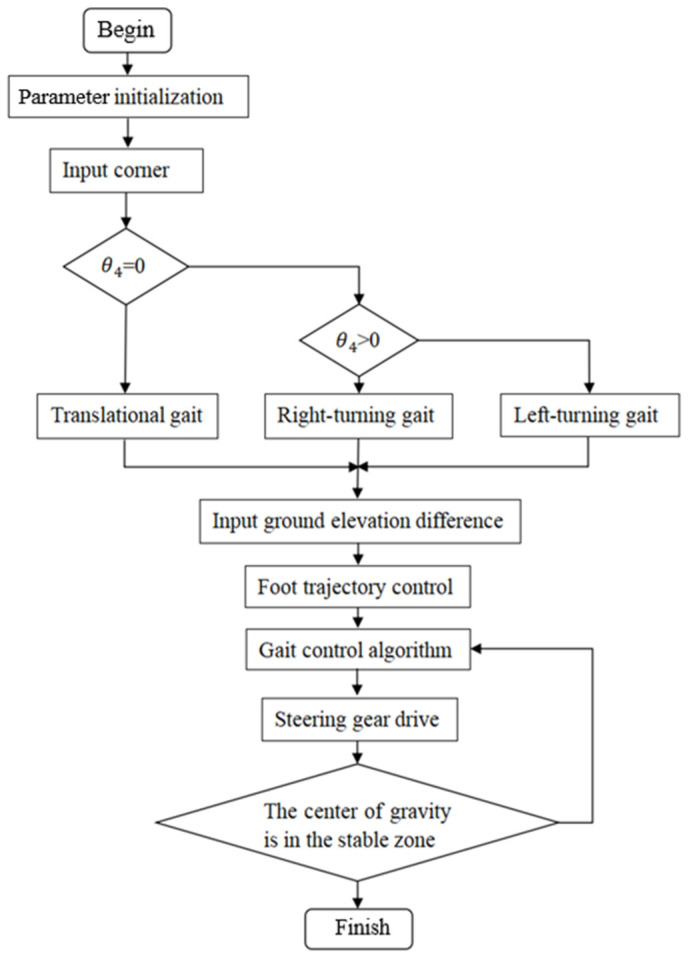
Block diagram of robot gait generation and control.

During the footed locomotion, the robot encounters unstructured and particularly rugged terrain. To achieve overall robot locomotion stability, the robot quantifies the unevenness of the road surface and sets thresholds based on different ranges of unevenness. When the unevenness is below the preset minimum threshold, the road surface is considered flat, and the robot moves with a gait adapted to the flat terrain. When the unevenness exceeds this threshold, the robot adjusts its gait according to the specific range of the unevenness and simultaneously regulates the joint movement to cross the obstacle. The specific gait control flow is illustrated in Figure 17, ensuring that the eight-legged wheel-legged robot can achieve stable movement in various complex terrains.

## 5. Simulation and Experimental Verification

A robot simulation model is established under the Adams and motion simulation environment to simulate and verify the robot’s various motion gaits. Simultaneously, an experimental platform is built to carry out the experimental verification of the robot’s different motion modes.

### 5.1. Simulation of Small Obstacle Movement on Flat Ground

**Figure 18:**
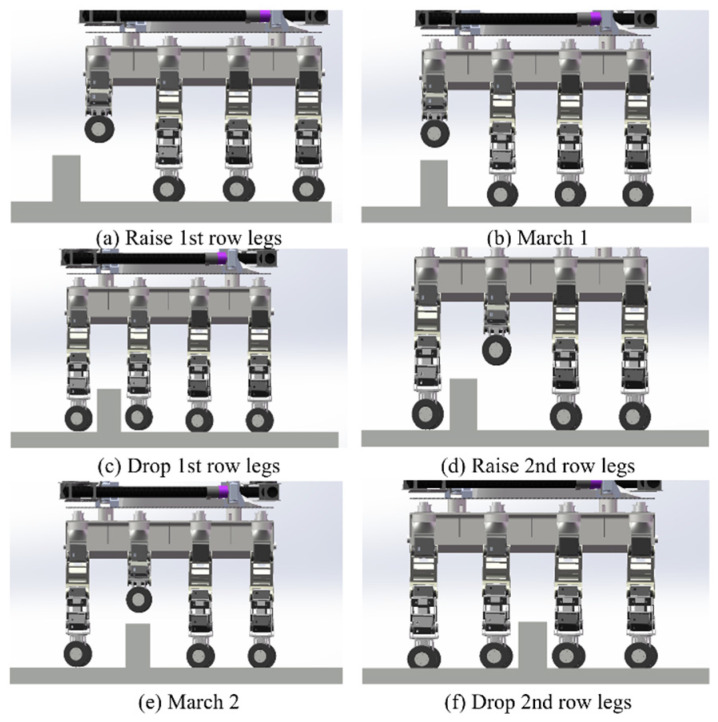
(**a**) Raise 1st row legs; (**b**) March 1; (**c**) Drop 1st row legs; (**d**) Raise 2nd row legs; (**e**) March 2; (**f**) Drop 2nd row legs. Small obstacle-crossing simulation.

**Figure 19:**
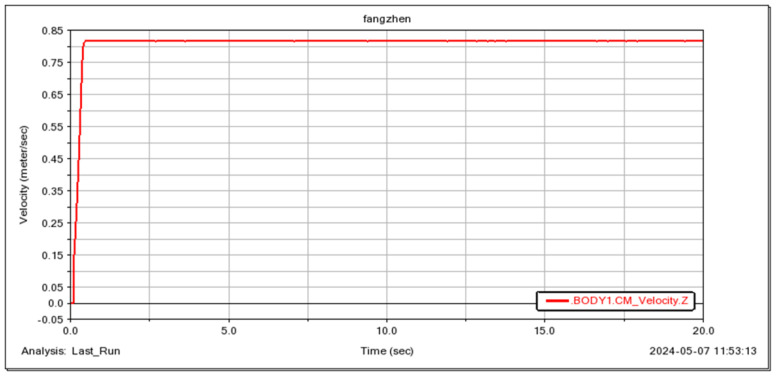
Robot movement speed.

**Figure 20:**
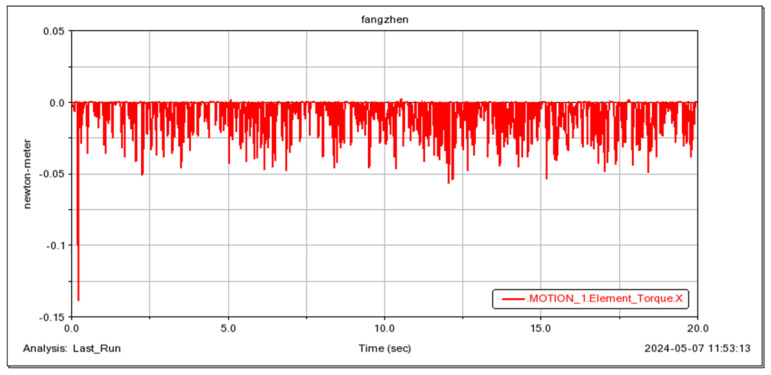
The driving torque of the robot’s wheels.

The robot’s ability to traverse small obstacles was simulated, and the results demonstrate the good passability of the wheeled mobility mode, as illustrated in Figure 18 and Figure 19. Simultaneously, the instantaneous driving torque of the wheel motors during the robot’s initial operation was verified. The results, shown in Figure 20, indicate that the wheel motors are subjected to a maximum torque of 0.14 N·m.

### 5.2. Going up the Steps

**Figure 21:**
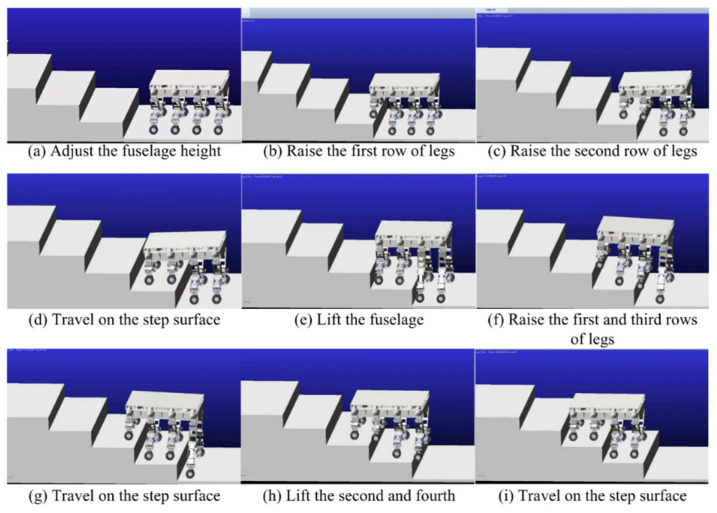
(**a**) Adjust the fuselage height; (**b**) Raise the first row of legs; (**c**) Raise the second row of legs; (**d**) Travel on the step surface; (**e**) Lift the fuselage; (**f**) Raise the first and third rows of legs; (**g**) Travel on the step surface; (**h**) Lift the second and fourth; (**i**) Travel on the step surface. Continuous step-up simulation test.

**Figure 22:**
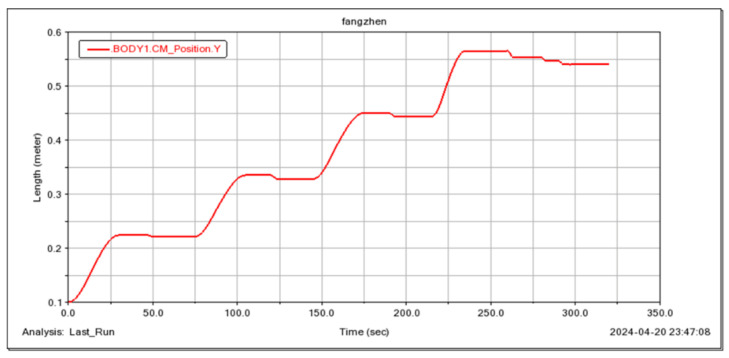
Robot center of mass height variation.

**Figure 23:**
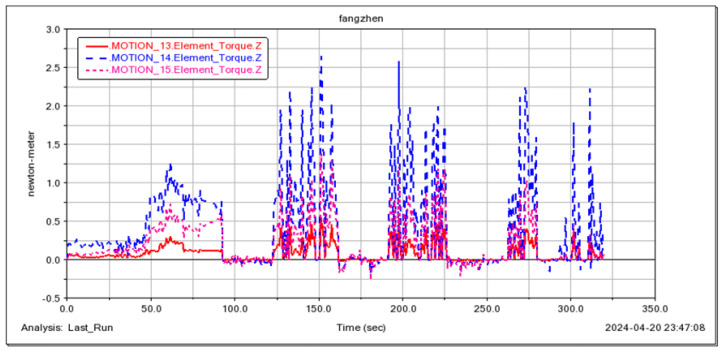
Driving torque of three servos in one leg.

**Figure 24:**
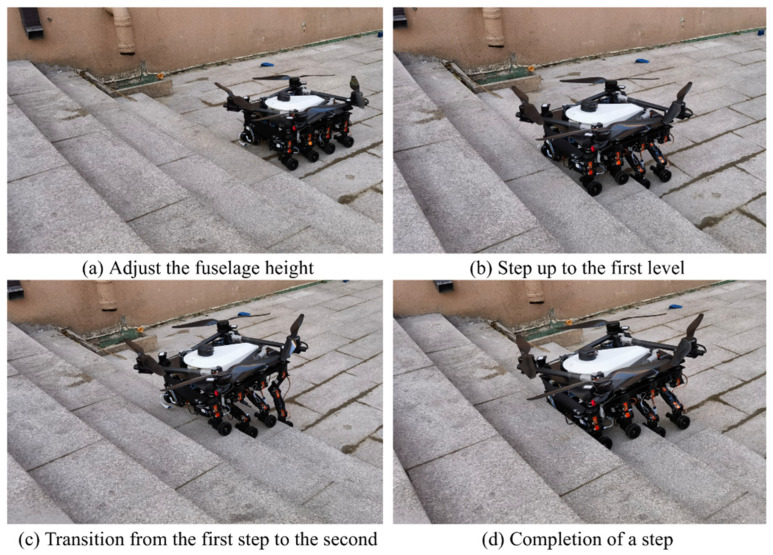
(**a**) Adjust the fuselage; (**b**) Step up to the first level; (**c**) Transition from the first step to the second; (**d**) Completion of a step. The 11.5 cm step test.

The feasibility of the robot for continuous step-ups was simulated and tested (Figure 21, Figure 22, Figure 23 and Figure 24). The simulation results show that the maximum torque of the leg servo does not exceed 2.7 N·m, which meets the servo torque requirements and verifies the correctness of the continuous step-up gait design. Taking the 11.5 cm high step as an example, the robot carried out the step-up test, and the test results were consistent with the simulation results.

### 5.3. Footwork

**Figure 25:**
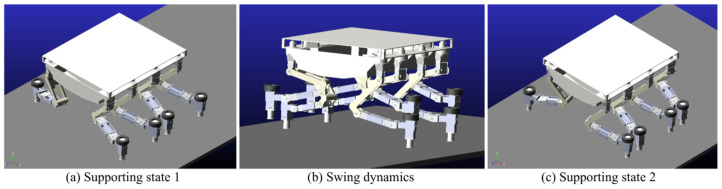
(**a**) Supporting state 1; (**b**) Swinh dynamics; (**c**) Supporting state 2. Simulation test of foot motion.

**Figure 26:**
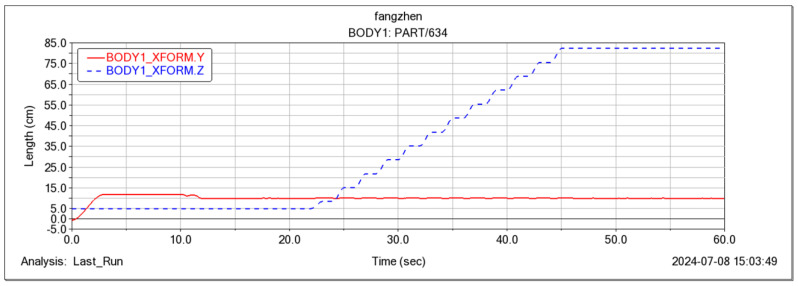
Foot motion center of mass position curve.

**Figure 27:**
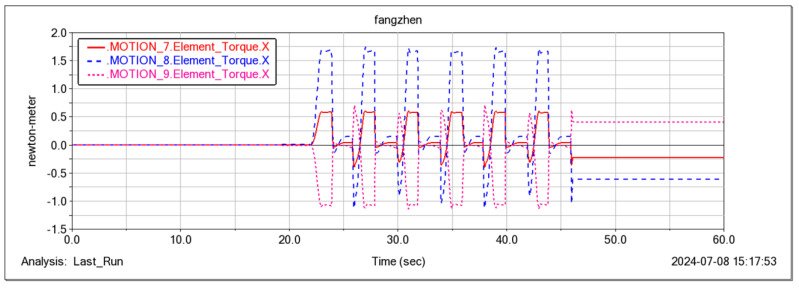
Moment curves of three servos around the X-axis for one leg.

**Figure 28:**
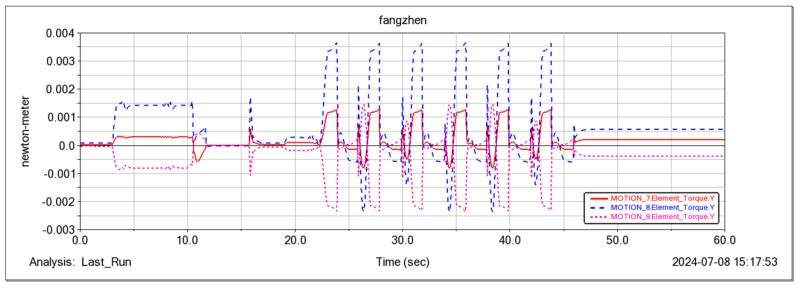
Moment curves of three servos around the Y-axis for one leg.

**Figure 29:**
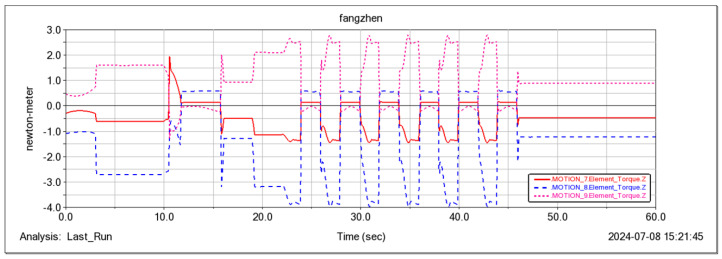
Moment curves of three servos around the Z-axis for one leg.

**Figure 30:**
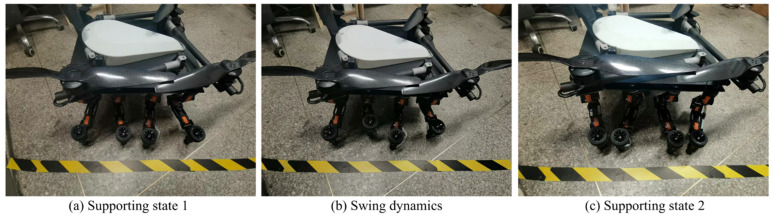
(**a**) Supporting state 1; (**b**) Swing dynamics; (**c**) Supporting state 2. Foot movement test.

In the Adams simulation environment (Figure 25), by monitoring the center coordinates of the fuselage, it was observed that the robot remained stable in the vertical direction (with a slight change of 10 cm) while moving forward periodically (Figure 26) using the gait described in Section 4.3. Concurrently, the torque in three directions of the three-jointed steering gears on one leg was monitored. It was noticed that the torque in each direction of the leg jointed steering gears changed periodically as the robot moved forward (Figure 26, Figure 27, Figure 28 and Figure 29), thereby verifying the stability and reliability of the gait. Through the field platform test (Figure 30), the research on the robot progressed smoothly, yielding results consistent with the simulation.

### 5.4. Flight Attitude

**Figure 31:**
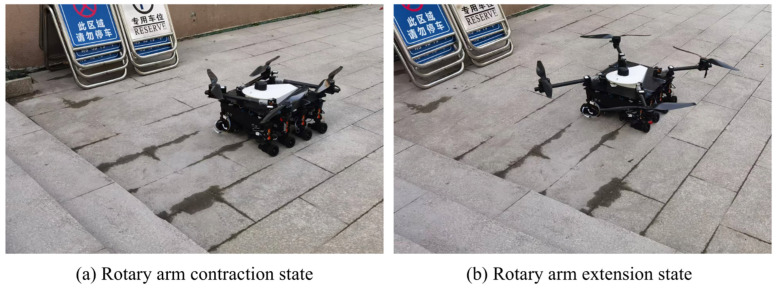
(**a**) Rotary arm contraction state; (**b**) Rotary arm extension state. Rotor deployment test.

The wheel-legged robot in this study is equipped with a self-designed and manufactured UAV system, featuring a rotating arm structure capable of dynamic adjustments. As illustrated in Figure 31, the contraction of the rotating arm during the ground movement enhances the robot’s trafficability. When ground locomotion becomes challenging, the robot system actively drives the cantilever structure to rotate along the direction of the built-in limit groove of the upper plate through the servo mechanism, enabling the attitude conversion from the ground walking mode to the flight mode. Furthermore, it can be observed that when the boom is fully extended to the working state, its mechanism design ensures that the space below the blade is completely free from interference with the fuselage part. This maximizes the utilization of the lift generated by the blade rotation, avoids the obstruction of the airflow by the fuselage, and consequently improves the overall aerodynamic efficiency and flight performance of the UAV. And during flight, all leg joints are contracted and pressed tightly against the fuselage to prevent vibrations during rotation from causing resonance and damage to the leg structure. Specifically, through the measurement of the ultrasonic module, the flight height of the UAV system can reach 5 m, and the endurance is 10 min, which further verifies the practical performance of the system in actual application scenarios.

## 6. Finite Element Analysis

**Figure 32:**
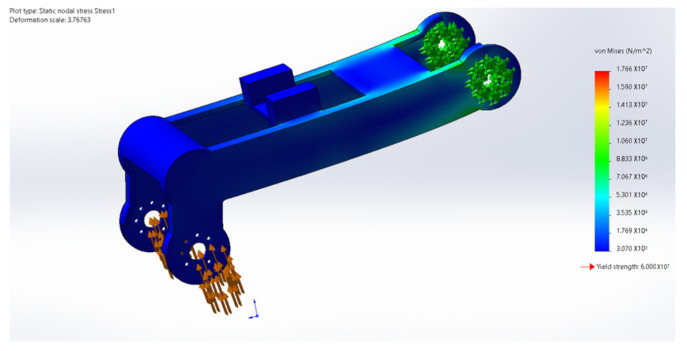
Thigh stress cloud.

**Figure 33:**
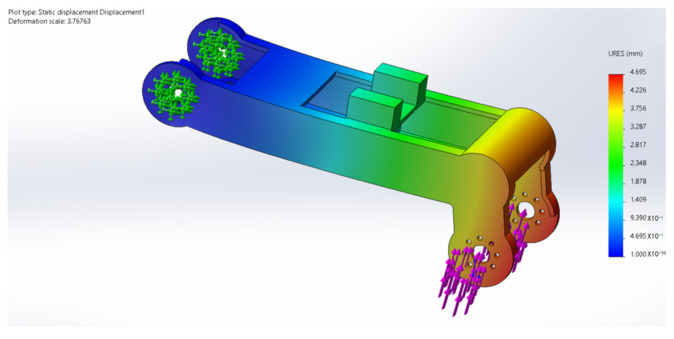
Thigh deformation cloud.

**Figure 34:**
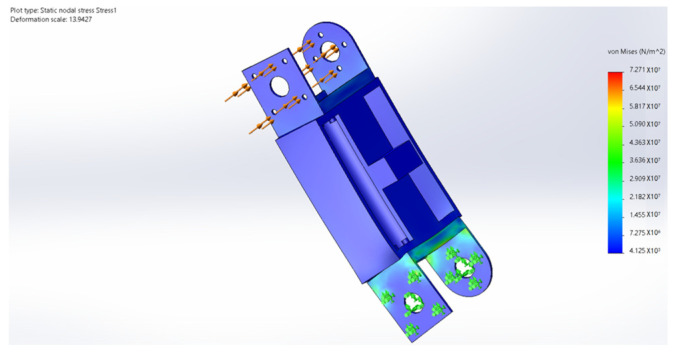
Calf stress cloud.

**Figure 35:**
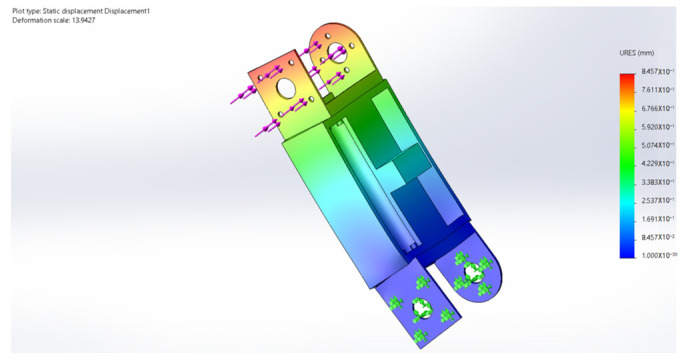
Calf deformation cloud.

**Figure 36:**
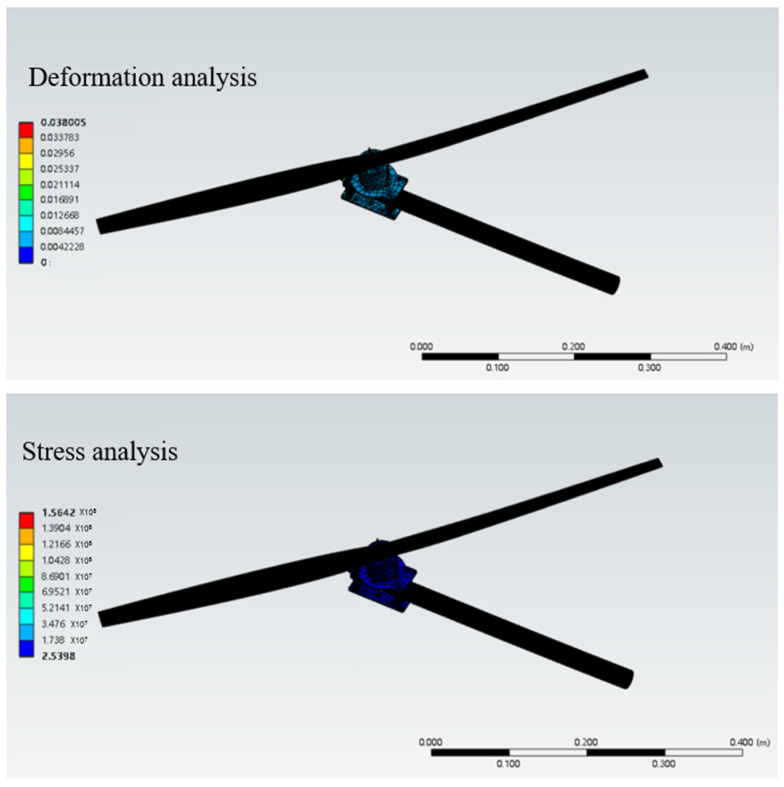
Finite element analysis of rotor unit.

During the movement of the eight-legged robot, the leg structure, including the thigh and calf, occasionally withstands substantial torque, necessitating high-strength requirements for the leg material. Additionally, the excessive mass of the leg structure would increase the burden on the brushless motors driving the servo and rotor. Through a quantitative comparison of engineering polymers, PA12 demonstrated a superior weight efficiency with its weight–strength ratio (49.0 MPa·cm^3^/g) being 58.3% lower than PA6-GF30 (117.6 MPa·cm^3^/g) while maintaining adequate strength for operational stresses. Crucially, its fatigue limit (25 MPa at 10^6^ cycles) exceeds the maximum cyclic stress amplitude observed during the gait motion (12–18 MPa), providing a safety factor of 1.39. Therefore, the main material selected is PA12, a suitable high-strength and lightweight material, while the leg structure undergoes lightweight design optimization. In terms of rotor units, carbon fiber was selected as the material, and its stress and deformation during movement were analyzed. Figure 32, Figure 33, Figure 34, Figure 35 and Figure 36 present the strength and stiffness checks of the main load-bearing parts to ensure the structural design’s rationality.

The analysis reveals that under the maximum loading condition, the maximum stress on the thigh of the eight-legged robot is 17.58 MPa, and the maximum deformation is 1.1 mm. These values fall within the acceptable range and will not compromise the mechanical structure or overall motion process.

The analysis indicates that under the maximum loading condition, the maximum stress on the leg of the eight-legged robot is 2.54 MPa, and the maximum deformation is 0.03 mm. These values are within the acceptable limits and will not affect the mechanical structure or overall motion process.

In the strength analysis of the rotor unit, the propeller speed was set to 600 rpm to simulate the lift required for actual hovering. The simulation showed that the stress on the entire cantilever unit was well below the yield strength of the material, ensuring stability during flight.

## 7. Conclusions

This study presents an innovative design of a highly adaptive octopedal wheel-legged robot capable of intelligently adjusting its wheel-leg configuration to navigate complex terrains. Through the application of an advanced kinematic analysis, simulation technology, and dynamic planning, the robot successfully implements and demonstrates a variety of gaits, including the ‘Step-Wise Octopedal Dynamic Coordination Gait’, showcasing an enhanced adaptability and mobility in challenging environments. The rigorous experimental validation, including field tests, confirms the effectiveness of the proposed design and control strategy, significantly improving the robot’s performance in tasks such as obstacle-crossing and step climbing. The platform design also accommodates an optional, integrated flight module with a functional deployment mechanism, providing a potential avenue for extending the operational reach in future applications. Crucially, the robot exhibits a significantly enhanced mobility across diverse and challenging terrains, a core capability addressed by this work. The insights gained from the kinematic modeling, gait planning, and experimental verification provide valuable engineering experience and data for the design and control of similar multi-modal robots. These results suggest the potential of such robots to contribute to applications in demanding scenarios like disaster rescue, exploration, and industrial automation, although further research and development are needed to fully realize this potential in real-world deployments.

## Figures and Tables

**Table 1:** Main parameters of the robot.

Fuselage Width (mm)	Fuselage Height (mm)	Same Row Leg Spacing (mm)	Fuselage Weight (kg)	Overrun Height (mm)
440	320	150	12	220

**Table 2:** Robot main dimensions and joint rotation angle limitations.

Listings	Numerical Value
di=1,4,5,8 (mm)	195.5
di=2,3,6,7 (mm)	204.2
$l_{i1wheel\;}(mm)$	106
$l_{i1leg\;}(mm)$	113
$l_{i2}\;(mm)$	134
$l_{i3}\;(mm)$	142.2
$l_{i4}\;(mm)$	142.2
$\;\theta_{i1\;}({}^{\circ})$	100~90
$\theta_{i2}\;({}^{\circ})$	60~170
$\theta_{i3\;}({}^{\circ})$	80~100
$\theta_{i4\;}({}^{\circ})$	80~100

## Data Availability

No new data were created or analyzed in this study.
